# Smokeless Tobacco Use Among Working Adults — United States, 2005 and 2010

**Published:** 2014-06-06

**Authors:** Jacek M. Mazurek, Girija Syamlal, Brian A. King, Robert M. Castellan

**Affiliations:** 1 Division of Respiratory Disease Studies, National Institute for Occupational Safety and Health, CDC; 2 Office on Smoking and Health, National Center for Chronic Disease Prevention and Health Promotion, CDC

Smokeless tobacco causes cancers of the oral cavity, esophagus, and pancreas (1). CDC analyzed National Health Interview Survey (NHIS) data to estimate the proportion of U.S. working adults who used smokeless tobacco in 2005 and 2010, by industry and occupation. This report describes the results of that analysis, which showed no statistically significant change in the prevalence of smokeless tobacco use among workers from 2005 (2.7%) to 2010 (3.0%). In 2010, smokeless tobacco use was highest among adults aged 25–44 years (3.9%), males (5.6%), non-Hispanic whites (4.0%), those with no more than a high school education (3.9%), and those living in the South (3.9%). By industry, the prevalence of smokeless tobacco use ranged from 1.5% in education services to 18.8% in mining industries, and by occupation from 1.3% in office and administrative support to 10.8% in construction and extraction. These findings highlight opportunities for reducing the health and economic burdens of tobacco use among U.S. workers, especially those in certain industries (e.g., mining) and occupations (e.g., construction and extraction) where use of smokeless tobacco is especially common. CDC recommends best practices for comprehensive tobacco control programs, including effective employer interventions, such as providing employee health insurance coverage for proven cessation treatments, offering easily accessible help for those who want to quit, and establishing and enforcing tobacco-free workplace policies (2).

NHIS is an annual, nationally representative, in-person survey of the noninstitutionalized U.S. civilian population. Questions about cigarette smoking are directed to one randomly selected adult in each surveyed family. In 2005 and 2010, data on cigarette smoking were collected from 31,428 and 27,157 persons, respectively. The same participants responded to a supplemental questionnaire that contained questions regarding the use of smokeless tobacco (i.e., chewing tobacco and snuff).* The survey response rates for the adult core and supplemental questionnaire combined were 69.0% in 2005 and 60.8% in 2010.

Survey participants were considered currently working if, when asked about their employment status during the week before their interview, they responded, “working at a job or business,” “with a job or business but not at work,” or “working, but not for pay, at a family-owned job or business.”† Information on participants’ current industry and occupation was coded by trained coders and grouped into 21 industry groups and 23 occupation groups.§ Current cigarette smokers were defined as respondents who reported having smoked ≥100 cigarettes during their lifetime and who reported currently smoking every day or some days. Current smokeless tobacco users were defined as respondents who reported having used chewing tobacco or snuff ≥20 times in their lifetime and who reported currently using chewing tobacco or snuff every day or some days. Dual users were defined as persons who were both current cigarette smokers and smokeless tobacco users. Sample weights were used to account for the complex sample design. Estimates with a relative standard error of ≥30% are not reported. Two-tailed t-tests were used to determine statistically significant differences between point estimates.¶

What is already known on this topic?

Smokeless tobacco use causes cancers of the oral cavity, esophagus, and pancreas. Smokeless tobacco use varies by age, sex, and education. Targeted workplace interventions are effective in reducing tobacco use.

What is added by this report?

Although current cigarette smoking among working adults was significantly lower in 2010 (19.1%) than in 2005 (22.2%), the prevalence of smokeless tobacco use among adult workers in 2010 (3.0%) did not significantly differ from 2005 (2.7%). The 3% prevalence in 2010 is 10 times the *Healthy People 2020* target of ≤0.3% for smokeless tobacco use among all U.S. adults. Smokeless tobacco use varied widely by industry and occupation, reaching 10.8% among construction and extraction workers. Among working adults who currently smoked cigarettes, the proportion who also used smokeless tobacco was 4.1% in 2005 and 4.2% in 2010.

What are the implications for public health practice?

These findings highlight opportunities for reducing the adverse health effects and economic impact of tobacco use among U.S. workers, especially those in certain industries (e.g., mining) and occupations (e.g., construction and extraction) where use of smokeless tobacco is especially common. CDC recommends best practices for comprehensive tobacco control, including effective employer interventions, such as providing employee health insurance coverage for proven cessation treatments, offering easily accessible help for those who want to quit, and establishing and enforcing tobacco-free workplace policies. Additionally, health-care providers can advise all their tobacco-using patients to quit.

The estimated number of adults aged ≥18 years who were working during the week before the interview was 141 million in 2005 and 139 million in 2010. Current smokeless tobacco use prevalence among working adults did not significantly differ from 2005 (2.7%) to 2010 (3.0%)** (p=0.87). The prevalence of smokeless tobacco use among working adults was highest among those aged 18–24 years (3.6%) in 2005 and those aged 25–44 years (3.9%) in 2010, and among males (4.9% and 5.6%, in 2005 and 2010, respectively), non-Hispanic whites (3.5% and 4.0%), those with no more than a high school education (3.6% and 3.9%), and those living in the Midwest (3.8% and 3.3%) or South (3.1% and 3.9%) (Table 1).

Current cigarette smoking prevalence among working adults aged ≥18 years was 22.2% in 2005 and 19.1% in 2010 (p<0.05).†† Among working adults who currently smoke cigarettes, the proportion who currently used smokeless tobacco (i.e., dual users) was 4.1% in 2005 and 4.2% in 2010 (p=0.55). In 2010, dual use was greatest among the following subgroups of working adult smokers: those aged 18–24 years (6.3%), males (7.3%), non-Hispanic whites (3.9%), those with no more than a high school education (4.5%), those with annual household income ≥$75,000 (4.8%), and those living in the Midwest (5.3%). Among adult workers, the average number of cigarettes smoked per day was significantly higher among dual users (15.5) compared with those who used cigarettes only (12.1) (p<0.05).

Reliable 2010 estimates of smokeless tobacco use were available for workers in 10 industry groups (Table 2). Prevalence of smokeless tobacco use in 2010 was highest among workers in mining (18.8%), wholesale trade (8.9%), and construction (7.9%) industries. Reliable estimates of dual use among smoking workers were available only for construction (10.2%) and manufacturing (7.1%) industries.

Reliable 2010 estimates of smokeless tobacco use were available for workers in eight occupation groups (Table 3). Prevalence of smokeless tobacco use in 2010 was highest among workers in construction and extraction (10.8%) and installation, maintenance, and repair (9.0%) occupations. No respondents in health-care support occupations reported smokeless tobacco use. Reliable estimates of dual use among smoking workers were available only for construction and extraction (14.5%) and production (5.7%) occupations.

## Discussion

In 2010, the prevalence of smokeless tobacco use among working adults (3.0%) exceeded the *Healthy People 2020* target of ≤0.3% for all U.S. adults, as did nearly all demographic and industry and occupation subgroups for which results are presented in this report. Although current cigarette smoking prevalence among working adults was significantly lower in 2010 (19.1%) than in 2005 (22.2%), the prevalence of smokeless tobacco use did not significantly differ from 2005 (2.7%) to 2010 (3.0%). The lack of reduction in smokeless tobacco use might be attributable to the introduction of novel smokeless tobacco products into the U.S. marketplace (e.g., snus and dissolvable tobacco), as well as increased expenditures§§ on smokeless tobacco marketing in recent years (3,4).

Tobacco industry advertising encourages cigarette smokers to use smokeless tobacco as an alternative in locations where smoking is not permitted (5,6). Additionally, research indicates that cigarette smokers might switch to smokeless tobacco for the purposes of harm reduction or smoking cessation (7). However, smokeless tobacco is not a safe alternative to combustible tobacco, and no conclusive scientific evidence currently exists showing that switching to smokeless tobacco promotes long-term cigarette smoking cessation (8). Because persons who concurrently use smokeless tobacco and cigarettes are less likely to report planning to quit than adults who smoke cigarettes exclusively (9), evidence-based interventions to reduce all forms of tobacco use are warranted. High-impact antitobacco media messages, comprehensive smoke-free policies, increased tobacco prices, and other interventions that prevent initiation and encourage cessation of tobacco products, in concert with sustained, comprehensive state tobacco control programs funded at CDC-recommended levels, are critical to decreasing tobacco use and reducing the health burden and economic impact of tobacco-related diseases in the United States (2).

The findings in this report are subject to at least four limitations. First, because tobacco use information was self-reported and was not validated by biochemical tests, the extent of underreporting or overreporting of tobacco use could not be determined. Self-reported current cigarette smoking status has been shown to have a high validity (10), but the validity of self-reported smokeless tobacco use has not been established. Second, limited sample size prevented the presentation of reliable estimates for some subpopulations. Third, the NHIS response rates of 69.0% and 60.8% might have resulted in nonresponse bias. Finally, the prevalence of smokeless tobacco use might be underestimated because certain smokeless tobacco products (e.g., snus) were not included in the NHIS questionnaire.

Health professionals can play an important role¶¶ in assessing smokeless tobacco use and advising users to quit. Results from this report identify industry and occupation groups with high prevalence of smokeless tobacco use where evidence-based cessation interventions could be effective in reducing tobacco use. Employers can help reduce tobacco use among employees by making their workplaces tobacco-free,*** providing employees with information on the health risks of tobacco use and the benefits of quitting, and sponsoring workplace-based tobacco cessation services, including employer-sponsored health insurance that covers proven treatments for tobacco use and dependence (2).††† Such efforts can help to achieve the *Healthy People 2020* objective to reduce smokeless tobacco use by adults to ≤0.3% by 2020.§§§

## Figures and Tables

**TABLE 1:** Current smokeless tobacco use,* cigarette smoking,† and proportion of cigarette smokers who use smokeless tobacco among working adults§ aged ≥18 years, by selected characteristics — National Health Interview Survey, United States, 2005 and 2010

Characteristic	2005 (N = 19,445)							2010 (N = 15,649)						
	
	Estimated¶ working population (1,000s)	Current smokeless tobacco use		Current cigarette smoking				Estimated¶ working population (1,000s)	Current smokeless tobacco use		Current cigarette smoking			
	
				%¶	(95% CI)	Proportion currently using smokeless tobacco					%¶	(95% CI)	Proportion currently using smokeless tobacco	
			
		%¶	(95% CI)			%¶	(95% CI)		%¶	(95% CI)			%¶	(95% CI)
**Overall**	**140,701**	**2.7**	**(2.3–3.1)**	**22.2**	**(21.4–22.9)**	**4.1**	**(2.9–5.2)**	**138,951**	**3.0**	**(2.7–3.5)**	**19.1**	**(18.3–19.8)**	**4.2**	**(3.3–5.0)**
**Age group (yrs)**														
18–24	18,355	3.6	(1.9–5.4)	26.4	(24.0–28.8)	—**	—	17,605	3.8	(2.6–5.3)	20.2	(17.8–22.5)	6.3	(3.0–9.6)
25–44	65,680	3.4	(2.9–3.9)	23.2	(22.2–24.3)	4.5	(3.3–5.6)	61,315	3.9	(3.3–4.7)	20.4	(19.2–21.7)	5.3	(3.9–6.8)
45–64	51,706	1.5	(1.2–1.9)	20.6	(19.5–21.7)	1.5	(0.8–2.3)	53,936	1.8	(1.4–2.3)	18.5	(17.2–19.7)	2.1	(1.1–3.0)
≥65	4,960	—	—	8.3	(6.3–10.3)	—	—	6,094	—	—	9.4	(7.1–11.7)	—	—
**Sex**														
Men	75,888	4.9	(4.3–5.6)	24.6	(23.5–25.7)	6.8	(4.9–8.7)	73,406	5.6	(4.9–6.3)	20.7	(19.6–21.8)	7.3	(5.8–8.8)
Women	64,813	—	—	19.3	(18.3–20.2)	—	—	65,544	—	—	17.4	(16.3–18.5)	—	—
**Race/Ethnicity**														
Hispanic	18,796	—	—	17.8	(16.4–19.3)	—	—	73,406	—	—	12.6	(11.2–14.1)	—	—
White, non-Hispanic	100,385	3.5	(3.0–4.0)	23.5	(22.6–24.4)	4.8	(3.3–6.3)	65,544	4.0	(3.5–4.6)	21.2	(20.2–22.2)	3.9	(3.4–4.5)
Black, non-Hispanic	15,146	—	—	19.7	(17.8–21.6)	—	—	73,406	0.9	(0.6–1.6)	18.1	(16.1–20.1)	0.9	(0.4–1.3)
Other	6,374	—	—	18.9	(15.6–22.2)	—	—	65,544	—	—	12.6	(10.2–15.0)	—	—
**Education**														
≤High school	54,652	3.6	(2.9–4.3)	30.8	(29.5–32.2)	4.7	(2.8–6.6)	47,605	3.9	(3.1–4.6)	28.3	(26.7–29.8)	4.5	(3.2–5.8)
>High school	84,901	2.1	(1.8–2.5)	16.6	(15.8–17.3)	3.3	(2.4–4.3)	90,927	2.6	(2.1–3.0)	14.4	(13.5–15.3)	3.8	(2.6–5.0)
Unknown	1,148	—	—	23.4	(16.0–30.8)	—	—	419	—	—	—	—	—	—
**Annual household income**														
$0—$34,999	23,127	2.6	(2.0—3.1)	30.6	(29.1–32.0)	3.9	(2.9–5.0)	31,905	2.7	(2.0–3.4)	28.6	(26.8–30.4)	3.8	(2.5–5.1)
$35,000—$74,999	43,279	3.4	(2.7—4.0)	23.7	(22.3–25.1)	4.2	(2.8–5.7)	45,281	3.0	(2.4–3.7)	21.3	(20.0–22.6)	4.2	(2.7–5.7)
≥$75,000	48,932	2.2	(1.7—2.8)	15.2	(14.0–16.5)	2.8	(1.3–4.4)	54,750	3.2	(2.5–3.8)	12.1	(11.1–13.2)	4.8	(2.6–7.0)
Unknown	25,363	2.5	(1.3—3.7)	19.7	(17.9–21.5)	—	—	7,015	—	—	16.9	(13.7–20.1)	—	—
**Health insurance**														
Not insured	24,662	2.8	(1.6–4.0)	32.9	(30.9–34.9)	—	—	24,151	3.0	(2.2–3.7)	31.4	(29.2–33.6)	4.1	(2.4–5.8)
Insured	115,545	2.7	(2.3–3.0)	19.9	(19.1–20.6)	3.9	(2.9–4.8)	114,221	3.0	(2.6–3.5)	16.6	(15.8–17.3)	4.2	(3.2–5.2)
Unknown	494	—	—	18.3	(6.6–30.0)	—	—	579	—	—	19.2	(3.2–35.2)	—	—
**Census region**														
Northeast	25,145	1.3	(0.7–2.0)	19.8	(18.0–21.5)	—	—	24,580	1.6	(0.9–2.3)	16.9	(15.0–18.8)	—	—
Midwest	36,465	3.8	(3.1–4.6)	26.0	(24.7–27.4)	4.6	(2.9–6.2)	33,168	3.3	(2.5–4.1)	21.6	(19.9–22.3)	5.3	(3.5–7.1)
South	49,831	3.1	(2.4–3.8)	23.1	(21.7–24.6)	4.7	(2.2–7.3)	48,802	3.9	(3.2–4.7)	21.0	(19.6–22.4)	4.2	(2.9–5.5)
West	29,260	1.7	(1.2–2.2)	17.7	(16.5–18.8)	3.1	(1.7–4.6)	32,401	2.4	(1.6–3.2)	15.6	(14.2–17.1)	3.7	(1.5–5.8)

**Abbreviation:** CI = confidence interval.

* Reported using chewing tobacco or snuff ≥20 times in their lifetime and currently using chewing tobacco or snuff every day or some days.

† Reported having smoked ≥100 cigarettes during their lifetime and currently smoking every day or some days.

§ Estimated number of adults who were employed during the week before interview.

¶ Weighted to provide national estimates using the survey sample weights for each participant.

** Estimates with a relative standard error ≥30% are suppressed

**TABLE 2:** Current smokeless tobacco use* and current cigarette smoking† among working adults§ aged ≥18 years, by industry — National Health Interview Survey, United States, 2005 and 2010

Industry	2005 (N = 19,445)					2010 (N = 15,649)				
	
	Estimated¶ working population (1,000s)	Current smokeless tobacco use		Current cigarette smoking		Estimated¶ working population (1,000s)	Current smokeless tobacco use		Current cigarette smoking	
			
		%¶	(95% CI)	%¶	(95% CI)		%¶	(95% CI)	%¶	(95% CI)
Agriculture, forestry, fishing, and hunting	2,187	8.8	(4.6–13.1)	16.6	(11.6–21.7)	2,019	—**	—	18.1	(12.5–23.7)
Mining	432	—	—	33.4	(18.0–48.8)	668	18.8	(7.9–29.7)	27.0	(15.9–38.2)
Utilities	1,298	—	—	20.7	(13.6–27.9)	1,353	—	—	18.2	(11.2–25.2)
Construction	11,245	6.4	(4.0–8.9)	33.4	(30.0–36.8)	9,253	7.9	(6.0–9.9)	29.5	(26.1–32.8)
Manufacturing	15,658	4.8	(3.5–6.0)	24.9	(22.7–27.1)	13,037	4.0	(2.9–5.2)	21.6	(19.2–24.0)
Wholesale trade	4,396	3.4	(1.7–5.1)	24.2	(20.1–28.2)	3,523	8.9	(5.0–12.9)	23.4	(18.3–28.5)
Retail trade	14,495	2.5	(1.6–3.5)	24.6	(22.2–27.1)	15,056	3.4	(1.9–4.9)	22.5	(19.9–25.1)
Transportation and warehousing	5,790	3.5	(1.9–5.1)	27.5	(23.6–31.4)	5,641	3.4	(1.5–5.2)	21.0	(17.4–24.5)
Information	3,199	1.8	(0.3–3.3)	20.8	(16.5–25.1)	3,433	—	—	15.6	(11.6–19.6)
Finance and insurance	6,726	—	—	16.5	(13.7–19.3)	5,970	—	—	14.5	(11.5–17.6)
Real estate and rental and leasing	2,876	—	—	25.8	(19.6–32.0)	2,643	—	—	23.6	(17.8–29.4)
Professional, scientific, and technical services	9,101	1.9	(0.9–2.8)	16.0	(13.8–18.3)	9,447	2.4	(1.2–3.6)	13.0	(10.8–15.2)
Management of companies and enterprises	27	—	—	—	—	84	—	—	—	—
Administrative and support and waste management and remediation services	5,319	3.6	(1.8–5.3)	28.6	(24.6–32.5)	6,037	4.1	(2.0–6.2)	25.1	(21.3–29.0)
Education services	12,880	0.8	(0.4–1.3)	10.6	(9.0–12.2)	13,835	1.5	(0.6–2.3)	8.5	(6.9–10.0)
Health care and social assistance	16,472	—	—	18.6	(16.6–20.6)	18,543	—	—	15.8	(13.9–17.8)
Arts, entertainment, and recreation	2,584	—	—	21.9	(17.0–26.8)	2,872	—	—	20.8	(14.9–26.7)
Accommodation and food services	7,644	—	—	34.0	(30.5–37.6)	9,090	—	—	31.1	(27.6–34.6)
Other services (except public administration)	6,504	—	—	20.3	(17.3–23.3)	7,112	—	—	17.1	(13.9–20.4)
Public administration	6,741	3.0	(1.5–4.6)	18.7	(16.1–21.4)	7,343	3.5	(2.0–5.0)	14.6	(12.1–17.2)
Armed forces††	64	—	—	19.6	—	127	—	—	14.0	(0.0–36.4)
Unknown	5,061	—	—	16.2	(12.9–19.4)	1,864	—	—	13.6	(8.2–18.9)

**Abbreviation:** CI = confidence interval.

* Reported using chewing tobacco or snuff ≥20 times in their lifetime and currently using chewing tobacco or snuff every day or some days.

† Reported having smoked ≥100 cigarettes during their lifetime and currently smoking every day or some days.

§ Estimated number of adults who were employed during the week before interview.

¶ Weighted to provide national estimates using the survey sample weights for each participant.

** Estimates with a relative standard error ≥30% are suppressed.

†† Includes only civilian employees; active military personnel excluded.

**TABLE 3:** Current smokeless tobacco use* and current cigarette smoking† among working adults§ aged ≥18 years, by occupation — National Health Interview Survey, United States, 2005 and 2010

Occupation	2005 (N = 19,445)					2010 (N = 15,649)				
	
	Estimated¶ working population (1,000s)	Current smokeless tobacco use		Current cigarette smoking		Estimated¶ working population (1,000s)	Current smokeless tobacco use		Current cigarette smoking	
			
		%¶	(95% CI)	%¶	(95% CI)		%¶	(95% CI)	%¶	(95% CI)
Management	13,082	2.2	(1.4–3.1)	17.3	(15.3–19.3)	13,434	3.3	(2.2–4.4)	12.7	(10.8–14.6)
Business and financial operations	5,880	—**	—	16.7	(14.1–19.4)	6,375	—	—	13.9	(10.9–16.9)
Computer and mathematical	3,029	—	—	12.8	(9.3–16.3)	4,028	—	—	12.2	(8.7–15.7)
Architecture and engineering	2,527	—	—	14.4	(9.7–19.0)	2,750	—	—	13.1	(8.8–17.5)
Life, physical, and social science	1,570	—	—	10.2	(5.0–15.4)	1,602	—	—	—	—
Community and social services	2,177	—	—	13.1	(8.5–17.6)	2,571	—	—	9.4	(6.0–12.9)
Legal	1,605	—	—	10.6	(6.0–15.3)	1,734	—	—	12.0	(7.0–17.1)
Education, training, and library	8,408	—	—	9.2	(7.5–10.9)	9,287	—	—	8.1	(6.1–10.2)
Arts, design, entertainment, sports, and media	2,546	—	—	17.9	(13.5–22.4)	2,847	—	—	13.3	(9.4–17.3)
Health-care practitioners and technical	6,908	—	—	13.6	(11.4–15.8)	6,876	—	—	10.3	(8.0–12.5)
Health-care support	2,954	—	—	23.5	(19.0–28.0)	3,436	—	—	28.5	(22.7–34.3)
Protective service	2,611	3.9	(1.7–6.1)	19.3	(14.8–23.9)	2,767	4.8	(2.2–7.5)	17.3	(12.5–22.0)
Food preparation and serving related	6,484	—	—	33.1	(29.2–36.9)	7,446	—	—	32.0	(28.0–36.0)
Building and grounds cleaning and maintenance	5,404	3.5	(1.9–5.0)	26.1	(22.2–30.0)	5,365	—	—	22.0	(18.2–25.9)
Personal care and service	3,916	—	—	23.8	(19.9–27.7)	5,001	—	—	16.4	(13.1–19.7)
Sales and related	14,507	1.9	(1.3–2.6)	24.0	(21.6–26.4)	14,277	3.6	(2.1–5.2)	22.0	(19.5–24.5)
Office and administrative support	18,988	1.1	(0.6–1.6)	20.8	(18.9–22.6)	18,102	1.3	(0.6–1.9)	18.4	(16.3–20.4)
Farming, fishing, and forestry	1,077	12.5	(5.3–19.8)	16.0	(9.4–22.7)	867	—	—	17.0	(7.6–26.5)
Construction and extraction	9,158	7.7	(4.6–10.8)	36.3	(32.6–40.0)	7,396	10.8	(8.2–13.4)	29.5	(25.6–33.3)
Installation, maintenance, and repair	4,832	5.1	(3.0–7.2)	29.5	(25.3–33.6)	4,904	9.0	(6.2–11.7)	28.9	(24.1–33.7)
Production	9,547	5.2	(3.2–7.1)	29.9	(27.0–32.7)	8,012	4.6	(2.8–6.4)	27.1	(24.0–30.2)
Transportation and material moving	8,305	4.6	(3.0–6.2)	31.9	(28.4–35.3)	7,789	4.5	(2.8–6.3)	28.7	(25.0–32.5)
Military††	79	—	—	—	—	133	—	—	—	—
Unknown	5,105	—	—	16.6	(13.4–19.8)	1,953	—	—	15.8	(9.9–21.7)

**Abbreviation:** CI = confidence interval.

* Reported using chewing tobacco or snuff ≥20 times in their lifetime and currently using chewing tobacco or snuff every day or some days.

† Reported having smoked ≥100 cigarettes during their lifetime and currently smoking every day or some days.

§ Estimated number of adults who were employed during the week before interview.

¶ Weighted to provide national estimates using the survey sample weights for each participant.

** Estimates with a relative standard error ≥30% are suppressed. Health-care support occupations was the only category for which no respondents using smokeless tobacco were identified.

†† Includes only civilian employees; active military personnel excluded.

## Footnotes

* Additional information available at http://www.cdc.gov/nchs/nhis/quest_data_related_1997_forward.htm.

† A total of 19,445 and 15,649 survey respondents were classified as currently working in 2005 and 2010, respectively.

§ Additional information available at ftp://ftp.cdc.gov/pub/health_statistics/nchs/dataset_documentation/nhis/2005/samadult_layout.pdf, http://www.census.gov/cps/files/occupation%20codes.pdf, and ftp://ftp.cdc.gov/pub/health_statistics/nchs/dataset_documentation/nhis/2008/naics_sectors_and_subsectors08.pdf.

¶ Additional information available at http://www.cdc.gov/nchs/data/series/sr_10/sr10_256.pdf.

** The age-adjusted smokeless tobacco use in 2010 was 2.9%. The estimate was age-adjusted to the 2000 U.S. standard population using the age groups 18–24, 25–34, 35–44, 45–64, and ≥65 years following the *Healthy People 2010* methodology (ftp://ftp.cdc.gov/pub/health_statistics/nchs/datasets/data2010/focusarea27/o2701b.pdf).

†† Additional information available at http://www.cdc.gov/mmwr/preview/mmwrhtml/mm6038a2.htm?s_cid=mm6038a2_w.

§§ Additional information available at http://www.ftc.gov/sites/default/files/documents/reports/federal-trade-commission-smokeless-tobacco-report-2011/130521smokelesstobaccoreport.pdf.

¶¶ Additional information available at http://bphc.hrsa.gov/buckets/treatingtobacco.pdf.

*** Tobacco-free workplaces allow no use of any tobacco products (including cigarettes, cigars, pipes, smokeless tobacco products, or of electronic cigarettes) by anyone at any time.

††† Additional information available at http://blogs.cdc.gov/niosh-science-blog/2010/12/10/smoking.

§§§ Additional information available at http://healthypeople.gov/2020/topicsobjectives2020/objectiveslist.aspx?topicid=41.
